# Prevalence and determinants of insomnia in patients with chronic heart failure: a cross-sectional survey in Jiaxing, China

**DOI:** 10.3389/fcvm.2026.1617201

**Published:** 2026-03-02

**Authors:** Haiqin Jin, Xiaoqin Men, Yan Sun, Zhihua Sheng, Lingsha Wu

**Affiliations:** Cardiovascular Department, The Second Hospital of Jiaxing, Jiaxing, Zhejiang, China

**Keywords:** chronic heart failure, cross-sectional survey, determinant, insomnia, prevalence

## Abstract

**Objective:**

To investigate the current status of insomnia in patients with chronic heart failure (CHF) and its influencing factors.

**Methods:**

Cross-sectional study,261 CHF patients who were hospitalized in the cardiovascular department of a tertiary-level hospital in Jiaxing City from December 2023 to August 2024 were included in the cross-sectional study, and were investigated by using a general information questionnaire, the Pittsburgh Sleep Quality Index (PSQI). The influencing factors were analyzed by univariate analysis and logistic regression using SPSS 26.0 software.

**Results:**

The prevalence of insomnia was 66.7% (174 patients) among 261 CHF patients.Logistic regression analysis showed that gender (OR = 13.566, 95%CI: 3.479∼52.899), mood (OR = 4.616, 95%CI: 1.266∼16.823), NYHA cardiac function classification (OR = 6.514, 95%CI: 2.475∼0.40417.142), chronic pain (OR = 6.334, 95%CI: 1.070∼37.475), per capita monthly household income (OR = 8.078, 95%CI: 2.870∼22.741), socialization range (OR = 4.312, 95%CI: 2.404∼7.732), smoking (OR =  3.673, 95%CI: 1.590∼8.317), number of comorbidities (OR = 3.698, 95%CI: 1.586∼8.622), and healthcare payment method (OR = 2.605, 95%CI: 1.290∼5.259) were the influential factor of insomnia in CHF patients.

**Conclusion:**

The prevalence of insomnia in CHF patients was high, and gender, mood, NYHA cardiac function class, chronic pain, per capita monthly household income, socialization range, smoking, number of comorbidities, and mode of healthcare payment were the influencing factors. It is important to note that cross-sectional study designs cannot establish causal relationships, but they can identify associated or coexisting risk factors.

## 1 Introduction

As a complex clinical syndrome, CHF is an end-stage manifestation of cardiovascular disease and the leading cause of death. The findings of 2017 revealed (1) that there are approximately 64.34 million patients with heart failure worldwide, with a prevalence of approximately 2% in adults, while in the elderly population, its prevalence climbs to more than 10%. The latest statistics reveal (2–4) that the growth in the number of CHF patients in China is the highest in the world, and the weighted prevalence of heart failure among residents aged ≥35 years is 1.3%; it is estimated that there are about 8.9 million existing heart failure patients in China, which undoubtedly creates heavy economic and psychological burdens on the society and families. Notably, about 30% to 40% of CHF is also accompanied by sleep disorders (5). This is due to the fact that CHF has a long duration of illness and is prone to recurrent episodes, and its characteristic pathological changes, such as low cardiac output, sleep apnea, and inappropriate use of diuretics, etc., not only cause physiological discomfort to patients, but also have a certain negative impact on their psychological health—patients are prone to anxiety, depression and other negative emotions, which will, to a certain extent, reduce the quality of sleep and lead to insomnia. These negative emotions can reduce the quality of sleep and lead to insomnia. Insomnia is a complication of CHF. Insomnia, in turn, has a negative impact on the recovery of cardiac function: difficulty in falling asleep increases patients' anxiety, depression and other negative emotions, which reduces their immune function and seriously affects their quality of life, and over time causes deterioration of cardiac function, gradually forming a vicious circle (6–8). Therefore, the prevention and treatment of insomnia in patients with CHF is urgent, and it is of great clinical significance to clarify the risk factors of insomnia. At present, clinical studies on the factors influencing insomnia symptoms are not consistent, and less attention has been paid to the CHF population. Based on this, the present study investigates the influencing factors of insomnia in CHF patients in order to provide a scientific theoretical basis for the prevention and treatment of insomnia.

## 2 Objects and methods

### 2.1 Objects

This study is a cross-sectional study. According to the sample size estimation method of Logistic regression analysis proposed by Chen Kun et al. (9): the sample size is 510 times the number of variables. This study contains a total of 25 assessment factors, the sample size should be: 25*(5∼10) = 125∼250 cases. Considering the 10% invalid questionnaires during data collection, the total sample size needed is 139∼278 cases. Using the convenience sampling method, 261 CHF patients hospitalized in the cardiovascular medicine department of a tertiary-level hospital in Jiaxing City were selected from December 2023 to August 2024 for the study. A total of 261 questionnaires were collected, and 261 questionnaires were valid, with a validity rate of 100%, which meets the sample size requirement.

Inclusion criteria: (1) Compliance with the Guidelines for Primary Care of Chronic Heart Failure (Practice Edition-2019) (10); (2) Age ≥18 years; (3) Those who voluntarily participated in this study and signed the informed consent. Exclusion criteria: (1) those who combined with other serious diseases or complications, such as severe hepatic and renal insufficiency, active malignant tumors, etc.; (2) those who had unclear thinking and cognitive dysfunction; (3) those who suffered from psychiatric diseases. Exclusion criteria: (1) those who filled out the questionnaire incompletely; (2) those who filled out the questionnaire randomly.

## 3 Methods

### 3.1 Development of the questionnaire form

The literature on the effects of insomnia in patients with chronic heart failure was searched in Chinese databases and guideline resource websites, such as the Chinese Journal Literature Database CNKI, Wanfang Database, and Wipro Full Text Database (VIP), as well as in English databases, such as PubMed, Cochrane Library, National Guideline Clearinghouse (NGC), EMBASE, EBSCO, and JBI Evidence-Based Health Care database and other English databases to search for literature on the factors affecting insomnia in patients with chronic heart failure. Two researchers independently screened the studies that met the criteria based on the inclusion criteria and exclusion criteria, and independently completed the quality evaluation of the included literature by applying the relevant quality evaluation tools to preliminarily form a questionnaire on the risk factors for insomnia in patients with chronic heart failure. The research group discusses the relevant topics of the included literature: members of the group analyze and discuss the results of the literature with their clinical practice experience, exchange opinions with each other, and if there is disagreement, vote by secret ballot so as to reach a consensus, so as to finalize the questionnaire on risk factors for insomnia in patients with chronic heart failure, which mainly includes age, gender, marital status, education, occupation, average monthly income, mode of medical payment, illness, and so on. monthly income, medical payment method, duration of illness, smoking and alcohol consumption, number of comorbidities, cardiac function class, mood, and chronic pain.

### 3.2 Questionnaire method

A questionnaire survey was conducted in face-to-face format. The researcher explains the purpose of the study to the patients, and after obtaining their informed consent, the researcher and the patients sign the informed consent form together. After the beginning of the survey, the researcher interpreted the content of the questionnaire using a unified guide, and it took about 15 min to fill in the questionnaire. After the completion of filling in the questionnaire, the researcher checked to make sure that there was no omission in the content of filling in the questionnaire, and then collected the questionnaire on the spot.

### 3.3 Insomnia judgment criteria

PSQI: compiled by foreign scholar Buysse in 1989, it is used to assess the sleep quality of patients in the last 1 month. This study employed a localized version of the PSQI scale to assess patients’ sleep quality and the Cronbach's *α* coefficient of the scale is 0.842, with 18 scoring entries, involving 7 dimensions of sleep quality, time to sleep, sleep duration, sleep disorders, sleep efficiency, hypnotic drugs, and daytime functioning (11). Each dimension is scored according to 0–3 points, with the total score ranging from 0 to 21 points, and the higher the score, the worse the sleep quality. In our country, PSQI > 7 indicates decreased sleep quality, and PSQI ≤ 7 indicates normal sleep quality.

### 3.4 Ethics approval and consent to participate

This study was reviewed by the Ethics Committee of The Second Hospital of Jiaxing (approval number 2023YJ005-01), and all patients gave informed consent and participated in the study voluntarily.

### 3.5 Statistical methods

Data were analyzed using SPSS 26.0 statistical software with a test level of *α*=0.05. General information of CHF patients and insomnia-related risk factors were statistically described using the number of cases and constitutive ratios, and the PSQI scores were described using the mean ± standard deviation ($\bar{x}$±s). Independent samples t-test or one-way ANOVA was used for univariate analysis of factors affecting insomnia in CHF patients; subsequently, multivariate logistic regression analyses were performed on statistically significant and clinically relevant variables.

## 4 Results

### 4.1 Basic information of CHF patients and incidence of insomnia

Insomnia was present in 174 (66.7%) of 261 CHF patients. The results of univariate analysis showed that gender, work status, family residence, occupational status, nature of occupation, residence, mood, NYHA cardiac function classification, chronic pain, monthly per capita family income, socialization range, smoking, alcohol consumption, number of comorbidities, comorbid diabetes mellitus, and healthcare payment method were all statistically significant (*P* < 0.05), as shown in Table 1.

**Table 1:** Comparison of the characteristics of insomniac and non-insomniac CHF patients [cases (%)].

Event	Insomniac (*n* = 174)	Non-insomniac (*n* = 87)	*F/t*	*P*
Gender			7.388	0.007
Men	82 (47.13)	60（68.97)		
Women	92 (52.87)	27（31.03)		
Working condition			4.501	0.035
Working	18 (10.34)	12（13.79)		
Retirement	156 (89.66)	75（86.21)		
Family residence			8.879	0.003
Village	74 (42.53)	28（32.18)		
Town	100 (57.47)	59（67.82)		
Occupational situation			3.482	0.016
Peasant	87 (50.00)	27 (31.03)		
Worker	64 (36.78)	45 (51.72)		
Profession	6 (3.45)	4 (4.60)		
Employees of enterprises and public institutions	17 (9.77)	11 (12.64)		
Nature of the occupation			4.621	0.011
Physical work	143 (82.18)	56 (64.37)		
Brain work	20 (11.49)	19 (21.84)		
Unbiased work	11 (6.32)	12 (13.79)		
Residential situation			4.438	0.013
Live alone	17 (9.77)	13 (14.94)		
Spouse or child	115 (66.09)	41 (47.13)		
Spouse and child	42 (24.14)	33 (37.93)		
Mood			40.934	<0.001
Anxieties	56 (32.18)	13 (14.94)		
Stabilise	118 (67.82)	74 (85.06)		
NYHA cardiac function classifications			23.749	<0.001
Level II	18 (10.34)	38 (43.68)		
Level Ⅲ	109 (62.64)	39 (44.83)		
Level Ⅳ	47 (27.01)	10 (11.49)		
Chronic pain			16.243	<0.001
Exist	30 (17.24)	5 (5.75)		
None	144 (82.76)	82 (94.25)		
Monthly per capita family income			48.829	<0.001
≤￥3,000	50 (28.74)	5 (5.75)		
￥3,000∼5,000	104 (59.77)	35 (40.23)		
≥￥5,000	20 (11.49)	47 (54.02)		
Social Scope			59.271	<0.001
Small	49 (28.16)	6 (6.90)		
Middle	78 (44.83)	8 (9.20)		
Large	21 (12.07)	47 (54.02)		
Larger	26 (14.94)	26 (29.89)		
Cigarette smoking			10.591	<0.001
Everyday	69 (39.66)	14 (16.09)		
Tobacco cessation	45 (25.86)	19 (21.84)		
Never	60 (34.48)	54 (62.07)		
Drinking wine			22.689	<0.001
Everyday	92 (52.87)	14 (16.09)		
Give up drinking	54 (31.03)	36 (41.38)		
Never	28 (16.09)	37 (42.53)		
Number of comorbidities			30.443	<0.001
≤1	35 (20.11)	50 (57.47)		
2-3	63 (36.21)	31 (35.63)		
≥4	76 (43.68)	6 (6.90)		
Combined diabetes			339.038	<0.001
Exist	97 (55.75)	9 (10.34)		
None	77 (44.25)	78 (89.66)		
Medical payment method			17.259	<0.001
New rural cooperative medical care	87 (50.00)	21 (24.14)		
Medical care for urban residents	43 (24.71)	16 (18.39)		
Employee medical insurance	44 (25.29)	50 (57.47)		

Mood was assessed using the Generalized Anxiety Disorder-7 Item Scale (GAD-7): a total score ≤4 indicated emotional stability, while a score >4 indicated anxiety. Chronic pain refers to persistent pain lasting ≥3 months. Social scope is assessed by evaluating the size and/or frequency of a patient's network of regular social interactions in daily life. Smoking cessation and alcohol abstinence refer to refraining from smoking and drinking for six months, as jointly attested by the patient and their family.

### 4.2 Logistic regression analysis results of insomnia in CHF patients

Logistic regression analysis was used to explore the influencing factors of insomnia in CHF patients. Taking whether insomnia occurred as the dependent variable, all the meaningful independent variables in Table 1 were included in the Logistic regression analysis, in which the continuous variables were substituted with the original values, and the categorical variables were assigned as shown in Table 2. The results showed that the likelihood ratio test of *P* < 0.01 indicated that the model passed the test, and among the goodness-of-fit, the Pearson *x*^2^ > 0.05 indicated that the goodness-of-fit was good. Gender, mood, NYHA cardiac function class, chronic pain, per capita monthly household income, socialization range, smoking, number of comorbidities, and healthcare payment method were associated with insomnia in patients with CHF, and the differences were statistically significant (*P* < 0.05), as shown in Table 3.

**Table 2:** Methods of assigning values to independent variables.

Independent variable	Description of the assignment
Gender (X_1_)	Women=1, men=2
Mood (X_1_)	Stabilise=1, anxieties=2
NYHA cardiac function classifications (X_3_)	Level Ⅱ=1, level Ⅲ=2, level Ⅳ=3
Chronic pain (X_4_)	None=1, exist=2,
Monthly per capita family income (X_5_)	≥￥5,000 = 1, ￥3,000∼5,000 = 2, ≤￥3,000 = 3
Social Scope (X_6_)	Larger=1, large=2, middle =3, small=4
Cigarette smoking (X_7_)	Never=1, tobacco cessation=2, everyday=3
Number of comorbidities (X_8_)	≥4 = 1, 2∼3 = 2, ≤ 1 = 3
Medical payment method (X_9_)	Employee medical insurance=1, medical care for urban residents=2, new Rural Cooperative Medical Care =3

**Table 3:** Multifactorial analysis of insomnia in patients with CHF (*n* = 261).

Event	B	SE	Wald x^2^	*P*	OR	95%CI
Gender	2.608	0.694	14.104	<0.001	13.566	3.479∼52.899
Mood	1.529	0.660	5.373	0.020	4.616	1.266∼16.823
NYHA cardiac function classifications	1.874	0.494	14.407	<0.001	6.514	2.475∼17.142
Chronic pain	1.846	0.907	4.141	0.042	6.334	1.070∼37.475
Monthly per capita family income	2.089	0.528	15.652	<0.001	8.078	2.870∼22.741
Social Scope	1.461	0.298	24.050	<0.001	4.312	2.404∼7.732
Cigarette smoking	1.291	0.422	9.356	0.002	3.673	1.590∼8.317
Number of comorbidities	1.308	0.432	9.167	0.002	3.698	1.586∼8.622
Medical payment method	0.957	0.358	7.134	0.008	2.605	1.290∼5.259

## 5 Discussion

### 5.1 Physiological psychology

Gender, chronic pain, and anxiety-depression states are significant risk factors for insomnia in CHF patients. In this study, the proportion of female patients with insomnia was significantly higher than that of males, consistent with existing research (12–14). This may be related to hormonal fluctuations, psychological characteristics, and higher family and social role pressures in women. Furthermore, chronic pain frequently co-occurs with sleep disturbances and emotional issues. CHF patients, burdened by long-term treatment stress, social limitations, and family caregiving responsibilities, are more prone to anxiety and depression, creating a vicious cycle between insomnia and psychological distress (15, 16). Therefore, clinical management of CHF should prioritize comprehensive mind-body interventions, integrating physiological treatments with psychological support.

### 5.2 Lifestyle

Cardiac dysfunction is a core factor affecting sleep in CHF patients. This study indicates that declining cardiac function is the primary cause of insomnia in CHF patients, with higher rates of insomnia occurring in those with poorer cardiac function. As cardiac function deteriorates, patients may develop pulmonary and systemic venous congestion, triggering symptoms such as coughing and dyspnea that severely disrupt sleep onset and maintenance. Additionally, CHF often coexists with sleep apnea, further disrupting sleep architecture. The study also indicates that the number of complications positively correlates with the severity of sleep disturbances. Insomnia may further exacerbate left ventricular remodeling and pulmonary hypertension, creating a vicious cycle. Regarding behavioral factors, smoking worsens insomnia through mechanisms including neural excitation, insufficient cerebral blood flow, airway irritation, and neurotransmitter disruption (17–21). Therefore, improving cardiac function, managing complications, and quitting smoking are key to enhancing patients' sleep quality.

### 5.3 Social factors

Social support serves as an independent protective factor against insomnia in CHF patients (*P* < 0.05). Stable economic income and healthcare coverage alleviate the psychological burden of medical expenses, thereby reducing anxiety and improving sleep (22). Family care and companionship help boost patient confidence and reduce psychological stress (23). Furthermore, extensive social networks alleviate feelings of loneliness, promote positive emotions, and collectively contribute to improved sleep quality. Therefore, establishing a comprehensive support system integrating family, medical, and social resources holds significant practical importance for CHF patients.

## 6 Limitations

There are some limitations to our study. First, the primary outcome was measured subjectively by a self-administered questionnaire, which may affect cerebral bias. Second, the survey was a face-to-face questioning survey by the researcher, and there may have been inconsistencies in the understanding of the survey in the interaction with the patients leading to erroneous results; additionally, underlying social desirability bias may also be present. Third, this study is subject to sample selection bias: we employed convenience sampling exclusively from our hospital, limiting its generalizability. Finally, as a cross-sectional study cannot help to determine the cause of the findings.

## 7 Conclusion

The incidence of insomnia among CHF patients is relatively high at 66.7%. Gender, emotional state, NYHA functional classification, chronic pain, per capita monthly household income, social network size, smoking status, number of comorbidities, and method of medical payment are independent factors influencing insomnia in CHF patients. Therefore, implementing targeted screening for key populations in clinical nursing can help concentrate limited nursing resources on those most in need, enhance preventive outcomes, and reduce the overall disease burden. Due to time constraints, this study did not implement intervention measures. At the same time, cross-sectional study designs cannot draw causal inferences. Future research is recommended to conduct longitudinal or intervention studies based on these findings to enrich knowledge in this field.

## Data Availability

The original contributions presented in the study are included in the article/Supplementary Material, further inquiries can be directed to the corresponding author/s.
